# Parametric Study of the Numerical Model of a Bolted Connection of Steel Structure for Photovoltaic Panels

**DOI:** 10.3390/ma15196794

**Published:** 2022-09-30

**Authors:** Damian Mrówczyński, Tomasz Gajewski, Tomasz Garbowski

**Affiliations:** 1 Doctoral School, Department of Biosystems Engineering, Poznan University of Life Sciences, Wojska Polskiego 28, 60-637 Poznań, Poland; 2 Institute of Structural Analysis, Poznan University of Technology, Piotrowo 5, 60-965 Poznań, Poland; 3 Department of Biosystems Engineering, Poznan University of Life Sciences, Wojska Polskiego 50, 60-627 Poznań, Poland

**Keywords:** parametric study, bolted connection, nutless screw, steel structures, photovoltaic panels, renewable energy sources

## Abstract

In the face of the reality that unexpectedly mobilized the governments of most central European countries (including Poland), the development of renewable energy sources (RES) seems to be an important direction. Therefore, both wind parks and solar farms will be constructed at double speed for energetic independence. This urgency makes the market of producers of structures for mounting solar panels also need to adapt quickly to the new situation. New constructions adapted to quick assembly with the use of nutless screw connections seem to be one of the best solutions. These structures must not only be easy and quick to install but also durable, which makes the connections resistant to cyclical loads. The speed of assembly of the substructure can be achieved precisely with the help of nutless connections, but their durability should be carefully analyzed. This article presents parametric analyses of the numerical model of this type of connection. The selection of appropriate numerical models for simulation is of key importance in the fatigue strength analysis of bolted connections. This article investigates two different models used in numerical fatigue analyses performed in the Abaqus FEA and FE-Safe program, namely, traditional bolt with nut and innovative self-tapping nutless bolt. Extended parametric analyses of both numerical models were carried out, which ultimately allowed optimization of the fatigue capacity of the connection.

## 1. Introduction

Despite globalization, energy independence is an important element of national energy policy. Its importance was shown in 2022 due to recent military conflict in eastern Europe, which has caused rapid growth in the price increase of fossil fuel, especially fossil gas. In this context, obtaining energy from renewable sources is an easy way to become independent of external energy suppliers and price changes in the market due to the political situation [1]. Moreover, the use of fossil fuels harms the environment, and their extraction is becoming increasingly more difficult due to the consumption of readily available deposits of fossil fuels [2].

For those reasons, obtaining energy from renewable sources is the current trend in developing and developed countries, not only in the European Union (EU) but also in Asia, South America, etc. According to a Eurostat report [3], “EU reached a 22.1% share of its gross final energy consumption from renewable sources in 2020”. For instance, Sweden has the biggest share of energy from renewable sources, i.e., about 60%. Three renewable energy sources count the most, i.e., wind, hydro, and solar power. Solar power production has grown the fastest in the EU: in 2008, the total electricity generated from solar sources was 1% while in 2020, it was 14% [3]. Two main types of solar power may be distinguished, namely, direct use of photovoltaic panels [4,5,6] and indirect use of concentrated solar power [7,8]. The first type has become very popular and can be run at a small scale (households, road signs, etc.) or large scale (commercial solar farms).

**Figure 1:**
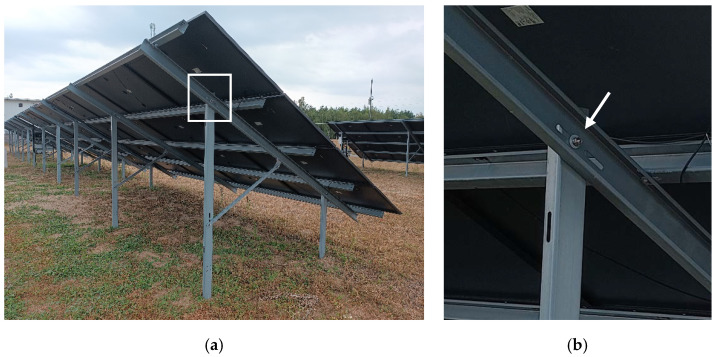
(**a**) An example of a substructure for photovoltaic panels made of thin-walled cross-sections with typical bolt connectors; (**b**) magnified bolt connector with a nut (source: grant POIR.01.01.01-00-0177/21).

**Figure 2:**
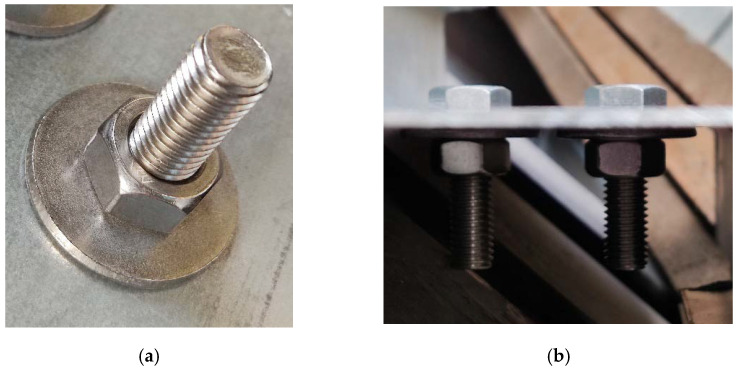
Pictures of the M12 bolt with hexagonal nut connectors: (**a**) view of the nut from the bottom of the joint and (**b**) side view (source: grant POIR.01.01.01-00-0177/21).

**Figure 3:**
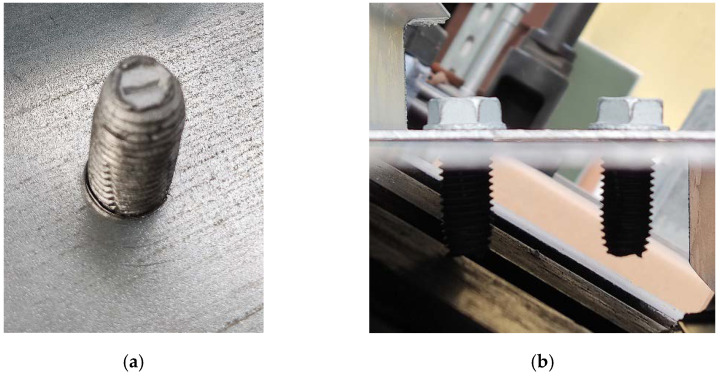
Pictures of self-tapping nutless connectors: (**a**) bottom view of the joint and (**b**) side view (source: grant POIR.01.01.01-00-0177/21).

Photovoltaic panels, regardless of whether they are rooftop or utility/ground mounted, require reliable and durable load-bearing supporting structures [9,10]. These types of structures are often exposed to extreme wind loads because they must be uncovered to eliminate, or at least minimize, the shadowing from surrounding objects. This effect is elevated in solar farms, in which large spaces are exposed to cyclic large wind loads and wind gusts. Therefore, the fatigue of the joints of the supporting structures must be taken into consideration. Often, the supporting structures are made of thin-walled sections, which serve as purlins, rafters, struts, and columns [11,12,13,14]. These types of steel members are light and adequate for use in shed-type structures [15,16]. The thin-walled structures are connected by bolt-type joints. In Figure 1, an example of the substructure for photovoltaic panels with the typical connectors is shown, i.e., bolts with nuts. In Figure 1, an overall view of the substructure on the solar farm is presented while in Figure 1, the magnified bolt connector with a nut is demonstrated. Here, the connector joins the thin-walled column with the thin-walled purlin. The most traditional bolt-type joints are screws with a hexagonal nut, as shown in Figure 2. Another interesting joint, however, not yet used on a daily basis, is the nutless connector with a self-tapping screw, as shown in Figure 3. Regardless of the type of bolt used, the fatigue analysis should be an inherent element of the design of the supporting structure of photovoltaic panel installations.

To verify novel steel connections, they can by modeled by the finite element method (FEM) and its results may be compared with the counterpart FEM results of traditional joints in terms of strength and fatigue analysis [17,18,19,20]. First, strength FEM analysis must be conducted, which provides the stress/strain field as input to a nominal advanced fatigue analysis. Numerical fatigue analysis is based on the analytical-empirical approach; however, it is performed for all nodes/elements of an FEM model. In numerical modeling, depending on the model simplifications, the results can differ by a dozen percent. Therefore, the details of the mathematical model included must be selected reasonably; too many details will greatly elevate the computational time while too many simplifications may burden the results with an unacceptably large error.

Scientists have devoted much attention to the development of models with acceptable accuracy and limited computational cost. For instance, in [21], a new finite element was proposed for the evaluation of load distribution and secondary bending. Its effectiveness was shown on a single-lap multi-bolt composite joint. High accuracy along with high computational savings were achieved. It may be particularly useful in a preliminary design to evaluate different configurations of the lap connection. As presented in [22], the use of advanced models may allow near real-time design decisions to be obtained in complex connections, such as multi-fastener composite bolted joints under various loading rates, including both static and dynamic load schemes. Less common loading protocols, such as fatigue, have also been studied to propose models for some typical connections to determine their strength, for instance, tubular joints strengthened with fiber-reinforced polymers [23,24]. These kinds of models require preliminary studies and model verification.

In this study, we analyzed the computational models of two types of joints, i.e., a bolt with a hexagonal nut connector and a self-tapping nutless connector. These bolts are presented in Figure 2 and Figure 3, respectively. By adopting the same numerical techniques and simplifications, we built duplicate models and assessed the fatigue strength of both connectors. Such analysis has not been published in the literature until now. Our aim was to answer the question of how the main modeling parameters influence the fatigue strength of both connectors and whether the new type of nutless connector has a similar fatigue strength to the traditional connector of the bolt with a nut. Our motivation is that the nutless connectors have not yet been fully experimentally verified and tested in real structures. Thus, numerical modeling is an excellent tool to conduct preliminary studies on nutless connectors. On the other hand, the self-tapping type of connectors are known and commonly used in wood structures [25,26] but are not yet popular in light steel structures. The new type of nutless connector has a very desirable feature in shed structures, namely, because they are self-tapping, their installation is much faster compared to structures with the bolt-nut connectors. Steel structures for photovoltaic panels would greatly benefit from this advantage of self-tapping nutless connectors.

## 2. Materials and Methods

### 2.1. Numerical Models and Parametrization

**Figure 4:**
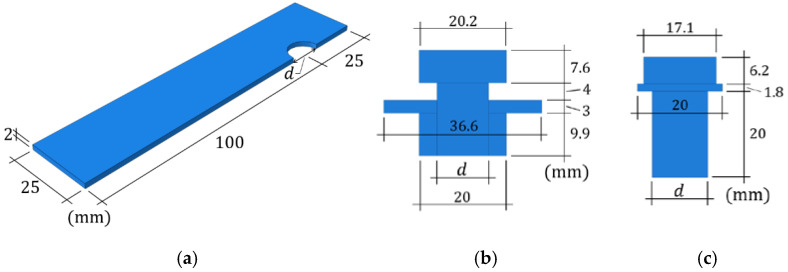
Geometries of the parts of the models: (**a**) plate; (**b**) prestressed bolt; (**c**) nutless bolt.

Numerical calculations were performed using FE commercial software (ABAQUS FEA [27]). Two types of lap connection were analyzed: (i) connection with the bolt and the nut and (ii) connection with the self-tapping nutless bolt. In order to reduce the computational time, only half of the lap connection was modeled with adequate boundary conditions instead of the entire geometry. In Figure 4, the geometries of the plates and bolts are shown. The variable d is the diameter of the bolt and the hole in the plate. This variable is parametrized, which means that it takes different values for different models.

**Figure 5:**
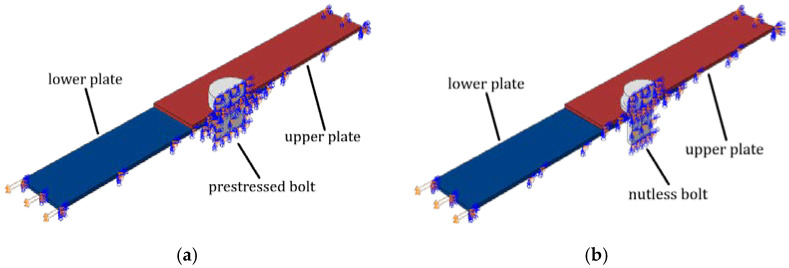
Boundary conditions for lap connections with (**a**) the prestressed bolt with a nut and (**b**) the nutless bolt.

The boundary conditions of the fixed support were applied on one end of the connection while at the other end, displacement was imposed, without the possibility to rotate the plate face. To obtain the appropriate behavior of the lap connection, the symmetry boundary conditions were defined to the cut plane (see Figure 5).

**Figure 6:**
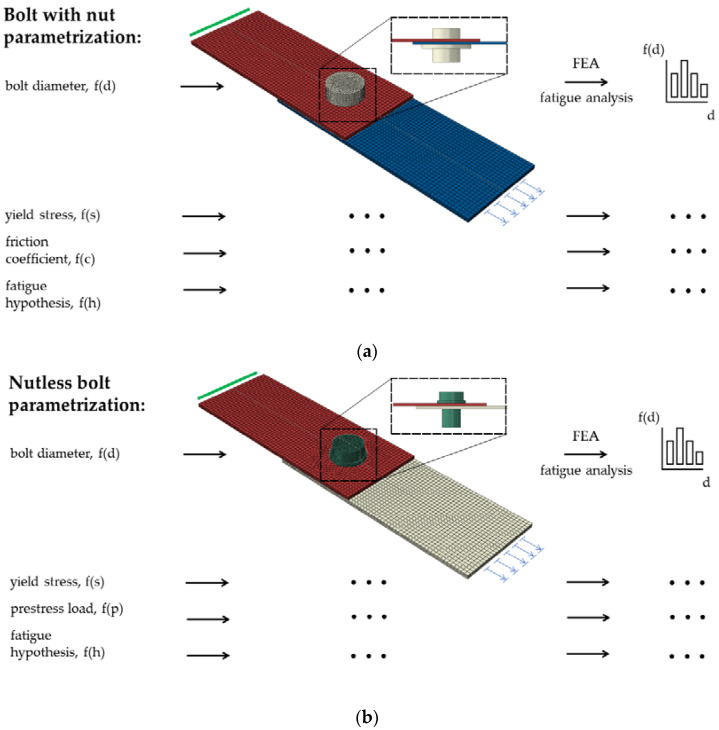
Schematic illustration of the fatigue study workflow: (**a**) parametrization for the bolt with a nut; (**b**) parametrization for the nutless bolt (schemes with the finite element meshes used).

For the connection with a bolt and a nut, the member was prestressed. In the first step, the bolt was prestressed and in the second step, the displacement was applied to the end of the plate. In the case of the nutless bolt connection, only one computational step was defined. The bolt sidewall was connected to the sidewall of the plate hole using the numerical technique to ensure structure integrity (continuity of displacements), the so-called ‘tie’ technique [27]. The main operating principle of this connection is to maintain the integrity of the structure at this point (no typical contact properties between the metal parts in contact); hence, the use of the applied connection is sufficient and reflects reality well. In both cases, the contact with basic Coulomb friction was defined between the contact surfaces, i.e., in the case of (1) nutless connection, between the plates and (2) prestressed bolt with a nut connection, between the plates and between the bolt sidewall and the sidewall of the plates. For all computations, 8-node linear brick and complementary 6-node linear triangular prism elements with full integration were used, labeled as C3D8 and C3D6 [27], respectively. For different sizes of bolts, different numbers of nodes were obtained. For example, for the nutless bolt, a reference model with a mesh size of 0.5 mm for the plates and 0.4 mm for the bolt was assumed, which gave 174,624 elements and 204,677 nodes. The finite element meshes used are shown in the schemes in Figure 6.

The models of both connections were subjected to parametric studies. Parametrization is a mathematical process consisting of expressing the state of a model as a function of some independent quantities called parameters. In this paper, the influence of parameters on the fatigue life of a prestressed lap connection with a nut and self-tapping nutless bolt was investigated. In the case of the bolt with a nut, the bolt diameter, yield strength, and prestressing force were parameterized, and various fatigue hypotheses were used (see Figure 6a). In the second case, the influence of the bolt diameter, yield strength, and coefficient of friction μ were examined. Likewise, various fatigue hypotheses were used (see Figure 6b). To capture the role of one variable by parametric studies, it is necessary to adopt a certain set of reference values of the parameters studied. The reference values of the parameters considered are presented in Table 1.

### 2.2. Fatigue Hypotheses

Fatigue analyses were performed based on the finite element method (FEM) and several fatigue hypotheses for ductile metals. It is often experimentally observed that a crack starts to form at the shear planes. Therefore, the maximum shear strain (MSS) criterion is one of the common fatigue hypotheses used:

(1) $$\frac{{\Delta \gamma }_{\max}}{2}=1.3\frac{\sigma_{f}^{\prime }}{E}{\left(2N_{f}\right)}^{b}+1.5\varepsilon_{f}^{\prime }{\left(2N_{f}\right)}^{c}\;,$$

where $\gamma_{\max}$ is the maximum shear deformation, $\varepsilon_{n}$ is the normal deformation to the maximum shear deformation, $\sigma_{f}^{\prime }$ is the fatigue strength coefficient, and $N_{f}$ is the fatigue strength.

According to the fatigue hypothesis of Brown–Miller [28], we postulate that the greatest fatigue failure occurs on the plane with the greatest shear strain and that it is a function of these shear strains and strains normal to this plane. If we include the Morrow mean stress correction [29] the Brown–Miller–Morrow (BMM) equation can be expressed as another commonly used criterion:

(2) $$\frac{{\Delta \gamma }_{\max}}{2}+\frac{{\Delta \varepsilon }_{n}}{2}=1.65\frac{\sigma_{f}^{\prime }-\sigma_{n,m}}{E}{\left(2N_{f}\right)}^{b}+1.75\varepsilon_{f}^{\prime }{\left(2N_{f}\right)}^{c}\;,$$

where $\varepsilon_{f}^{’}$ is the fatigue ductility coefficient and $\sigma_{n,m}$ is the mean in-plane normal stress.

The Morrow mean stress correction [29] takes the following form:

(3) $$\frac{{\Delta \varepsilon }_{n}}{2}=\frac{\sigma_{f}^{\prime }-\sigma_{n,m}}{E}{\left(2N_{f}\right)}^{b}+\varepsilon_{f}^{\prime }{\left(2N_{f}\right)}^{c}\;.$$

Another interesting hypothesis includes the Smith–Watson–Topper (SWT) mean stress correction [30]. The authors proposed that the fatigue life is a function of the product of the strain amplitude and the maximum stress in the cycle, namely:

(4) $$\frac{\Delta \varepsilon }{2}\sigma_{\max}=\frac{{\left(\sigma_{f}^{\prime }\right)}^{2}}{E}{\left(2N_{f}\right)}^{2b}+\sigma_{f}^{\prime }\varepsilon_{f}^{\prime }{\left(2N_{f}\right)}^{b+c}\;.$$

In this paper, the fatigue analyses were performed in the Abaqus FEA and FE-Safe programs from Dassault Systemes. The Abaqus FEA program was used to perform a nonlinear strength analysis in order to calculate the stress and deformation fields for two types of connections considered (bolt with a nut and nutless bolt). The FEA outputs were the input stress/strain fields for the FE-Safe program, in which the nominal fatigue analyses were performed according to four different hypotheses: the maximum shear strain criterion (Equation (1)), the Brown–Miller–Morrow criterion (Equation (2)), the Morrow criterion (Equation (3)), and the Smith–Watson–Topper criterion (Equation (4)).

Due to the fact that the orientation of the principal strains/stresses can change during loading, the computations include the algorithm for searching for the critical plane. This algorithm calculates the failure in planes with an increment of 10°. In the fatigue analysis, the load signal was adopted as the sine load from the FEM analysis.

## 3. Results

### 3.1. Prestressed Bolt with Nut Connection

#### 3.1.1. Bolt Diameter

**Figure 7:**
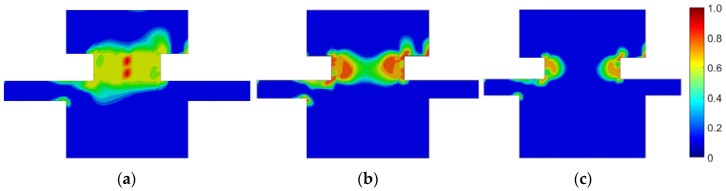
Fatigue distribution of the normalized logarithmic life repeats in the bolt for the model with a prestressed bolt with a nut diameter of: (**a**) 10 mm; (**b**) 12 mm; and (**c**) 14 mm.

**Figure 8:**
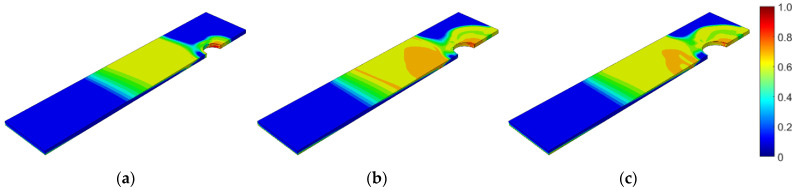
Fatigue distribution of the normalized logarithmic life repeats in the lower plate for the model with a prestressed bolt with a nut diameter of: (**a**) 10 mm; (**b**) 12 mm; (**c**) 14 mm.

**Figure 9:**
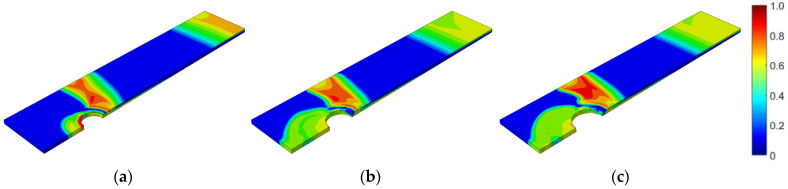
Fatigue distribution of the normalized logarithmic life repeats in the upper plate for the model with a prestressed bolt with a nut diameter of: (**a**) 10 mm; (**b**) 12 mm; (**c**) 14 mm.

**Figure 10:**
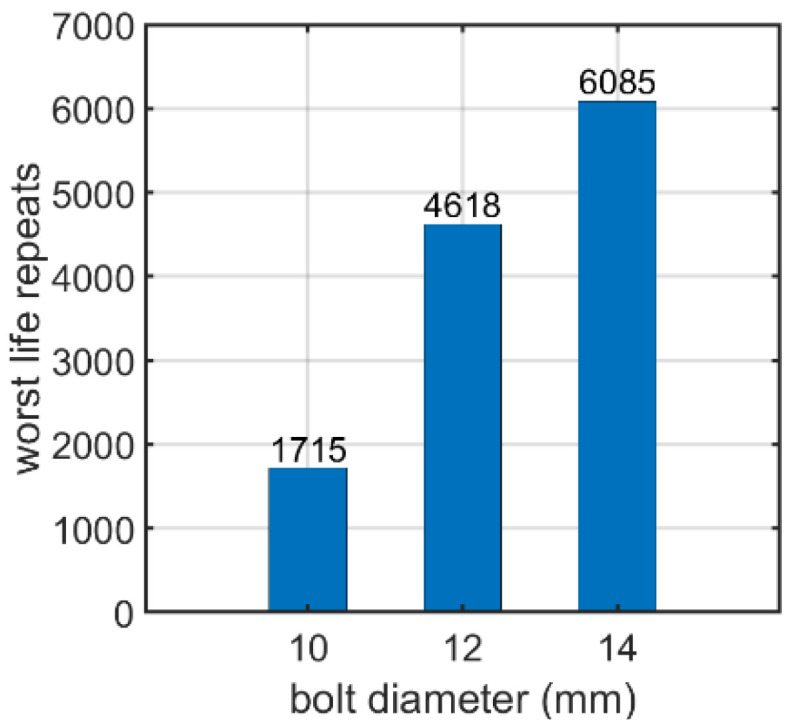
Number of logarithmic life repeats for diameter parameterization of the prestressed bolt with a nut.

First, the prestressed bolt with a nut connection was analyzed. In this case, the influence of the bolt diameter, bolt yield stress, prestressing force, and fatigue hypothesis on the number of logarithmic life repeats was investigated. Bolt diameters that are commonly used in photovoltaic structures were analyzed: 10, 12, and 14 mm. After static computations, the fatigue analyses were performed. Figure 7, Figure 8 and Figure 9 present the field distributions of the logarithmic life repeats obtained for different parts of the prestressed bolt connection and the analyzed models. The structure will endure $10^{n}$ repeats, in which n is the logarithmic life repeats. In Figure 10, the logarithmic life repeats for bolt diameter parametrization are shown, i.e., the minimal values taken for each connection from each fatigue distribution of the logarithmic life repeats (Figure 7, Figure 8 and Figure 9).

#### 3.1.2. Yield Strength of the Bolt

**Figure 11:**
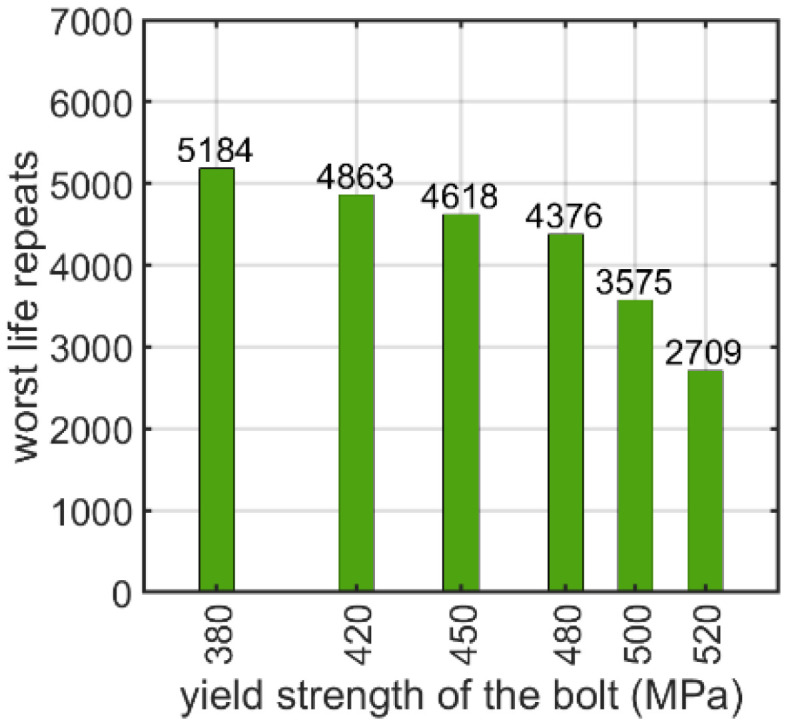
Number of logarithmic life repeats for parameterizing the yield stress of the prestressed bolt with a nut.

The next parameter tested was the yield strength of the bolt with a nut. In this case, yield strengths corresponding to commonly used steel grades and several intermediate values were adopted. Figure 11 shows the number of logarithmic life repeats for the yield strength parametrization. The values presented are the minimal values of the logarithmic life repeats taken for each connection from the fatigue distribution (not shown).

#### 3.1.3. Prestressing Force

**Figure 12:**
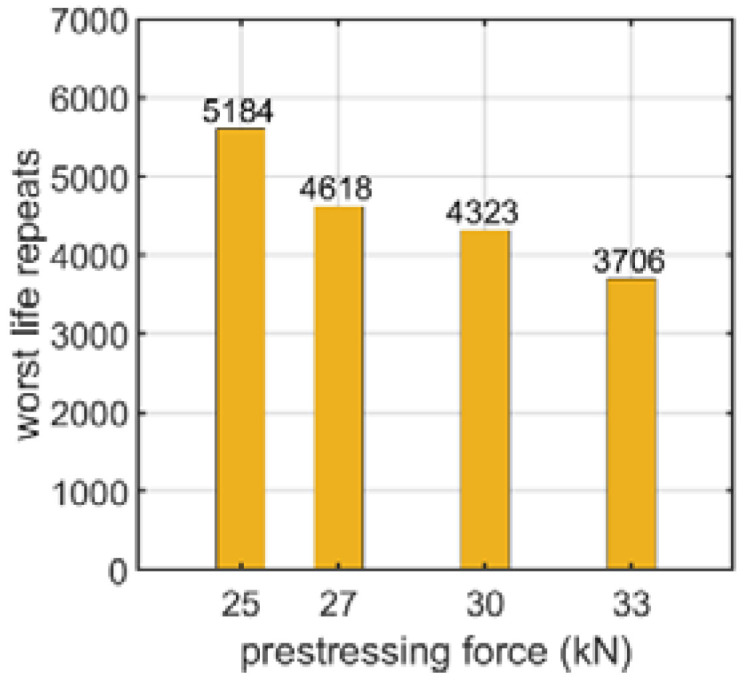
Number of logarithmic life repeats for prestressing force parametrization of the bolt with a nut connection.

Another factor analyzed was the prestressing force in the bolt with a nut. The prestressing force was applied in the first step of the static analysis and the displacement was applied in the second step (see Section 2.1). Then, the fatigue analysis was performed, from which the influence of the prestressing force on the lap connection fatigue life was obtained (see Figure 12). The values presented are the minimal values of the logarithmic life repeats taken for each connection from the fatigue distribution (not shown).

#### 3.1.4. Fatigue Analysis Algorithm

**Figure 13:**
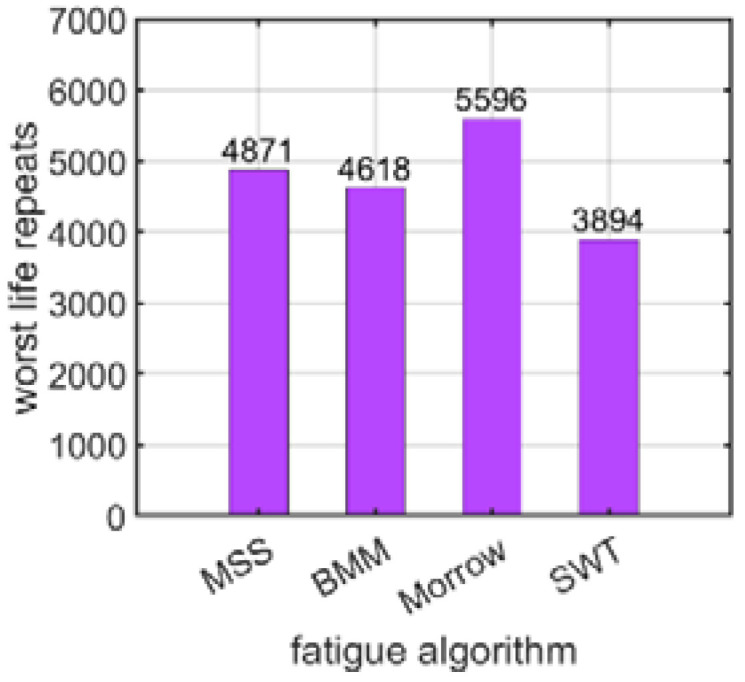
Number of logarithmic life repeats of the prestressed bolt with a nut connection for different fatigue algorithms.

The last factor analyzed was the fatigue algorithm. Four fatigue hypotheses were adopted here: the maximum shear strain criterion (MSS), the Brown–Miller–Morrow criterion (BMM), the Morrow criterion (Morrow), and the Smith–Watson–Topper criterion (SWT), all of which are described in Section 2.2. In Figure 13, the results of the logarithmic life repeats for different fatigue algorithms for the prestressed bolt with a nut are presented. The values presented are the minimal values of the logarithmic life repeats taken for each connection from the fatigue distribution (not shown).

### 3.2. Nutless Bolt Connection

#### 3.2.1. Bolt Diameter

**Figure 14:**
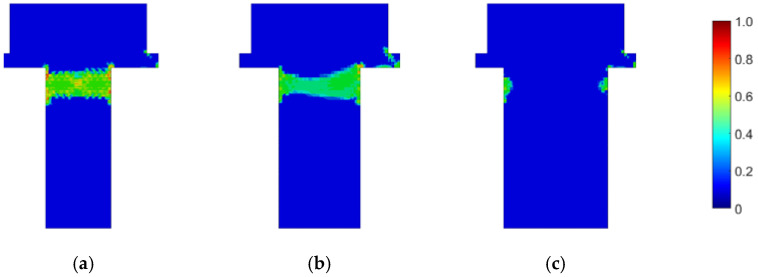
Fatigue distribution of the normalized logarithmic life repeats in the nutless bolt for the model with a bolt diameter of: (**a**) 8.2 mm; (**b**) 10.2 mm; (**c**) 13 mm.

**Figure 15:**
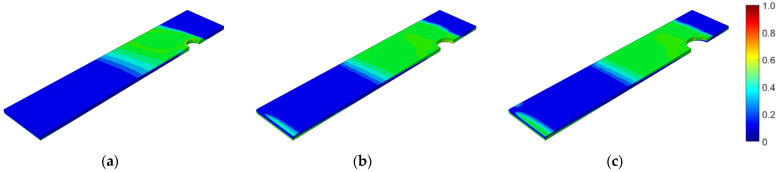
Fatigue distribution of the normalized logarithmic life repeats in the lower plate for the model with a bolt diameter of: (**a**) 8.2 mm; (**b**) 10.2 mm; (**c**) 13 mm.

**Figure 16:**
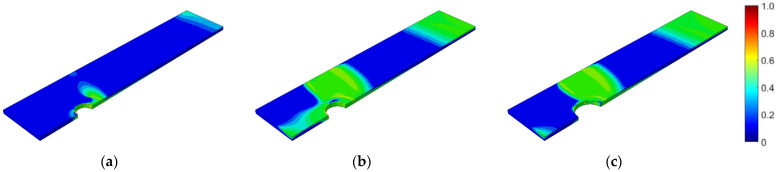
Fatigue distribution of the normalized logarithmic life repeats in the upper plate for the model with a bolt diameter of: (**a**) 8.2 mm; (**b**) 10.2 mm; (**c**) 13 mm.

**Figure 17:**
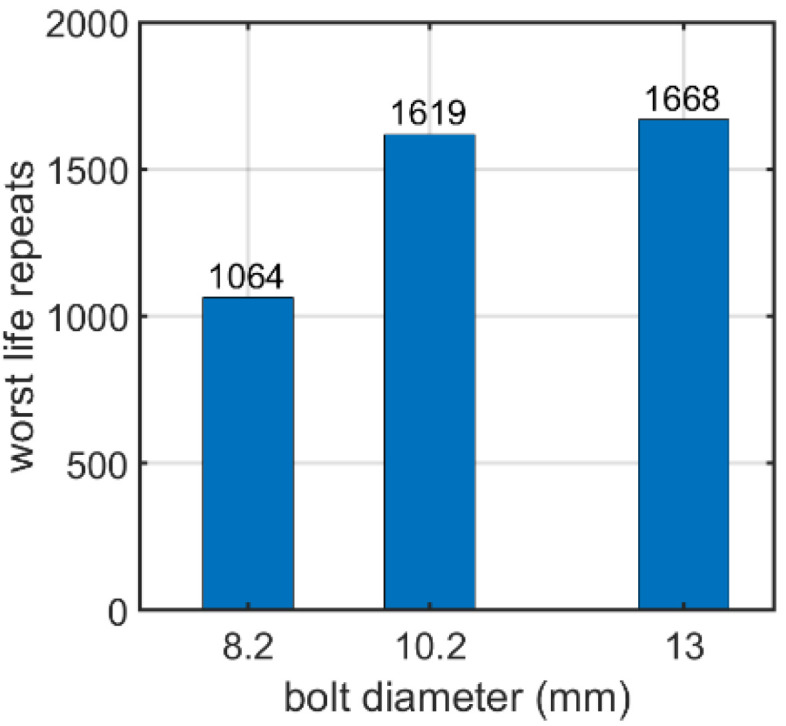
Number of logarithmic life repeats for diameter parameterization of the nutless bolt.

Second, the self-tapping nutless bolt connection was analyzed. In this case, the bolt diameter, bolt yield stress, coefficient of friction, and applied fatigue algorithm were parameterized. In terms of the bolt diameter, connections with three different bolt diameters were modeled: 8.2, 10.2, and 13.0 mm. The adopted diameters correspond to the bolts available from the manufacturer. After the static computations, fatigue analyses were performed. Figure 14, Figure 15 and Figure 16 show the field distribution of the logarithmic life repeats for different parts of the connection of the analyzed models. In Figure 17, the logarithmic life repeats for different bolt diameters are presented.

#### 3.2.2. Yield Strength of the Bolt

**Figure 18:**
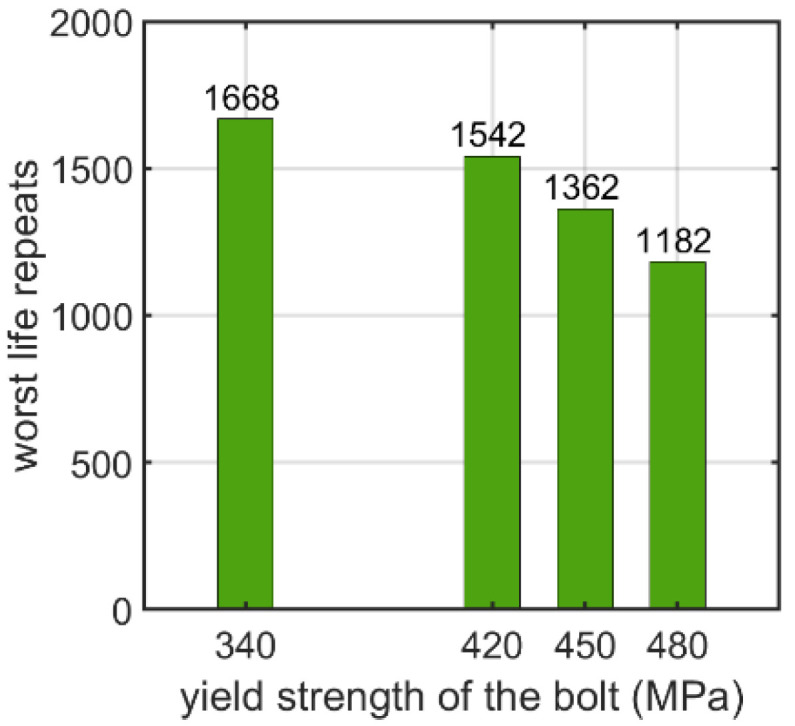
Number of logarithmic life repeats for parameterizing the yield stress of the nutless bolt.

The yield strength of the nutless bolt was also tested as a parameter. In this paper, yield strengths corresponding to commonly used steel grades were assumed. The influence of the yield strength on the number of logarithmic life repeats is shown in Figure 18. The values presented are the minimal values of the logarithmic life repeats taken for each connection from the fatigue distribution (not shown).

#### 3.2.3. Coefficient of Friction

**Figure 19:**
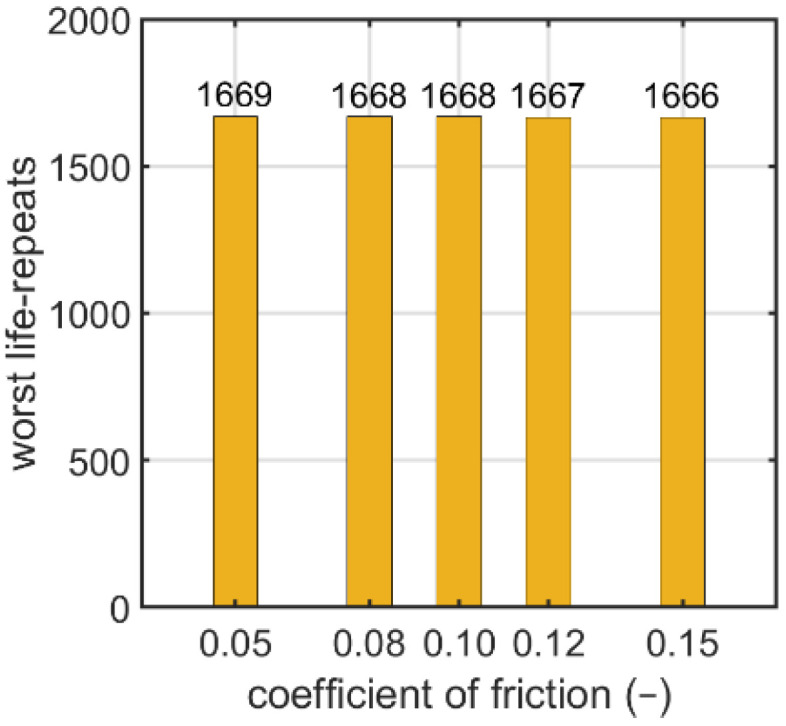
Number of logarithmic life repeats for parametrizing the coefficient of friction of the nutless bolt.

Another parameter analyzed was the coefficient of friction. The contact conditions for the nutless bolt connection are described in Section 2.1. In this case, five values of the coefficient of friction were analyzed: 0.05, 0.08, 0.10, 0.12, and 0.15. In Figure 19, the logarithmic life repeats obtained for the analyzed parameters are presented. The values presented are the minimal values of the logarithmic life repeats taken for each connection from the fatigue distribution (not shown).

#### 3.2.4. Fatigue Analysis Algorithm

**Figure 20:**
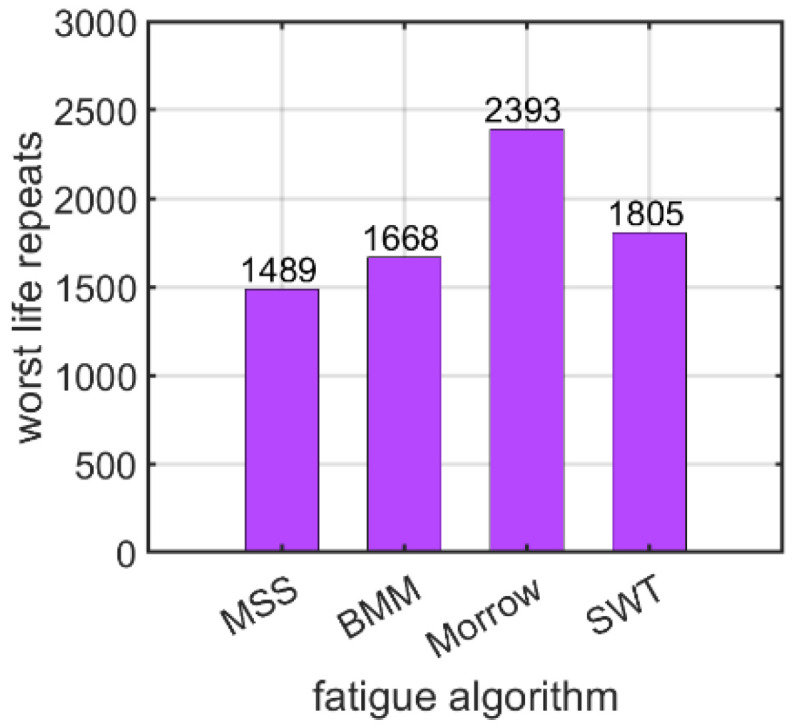
Number of logarithmic life repeats of the nutless bolt connection for the different fatigue algorithms used.

The last factor was the choice of the fatigue algorithm. The fatigue hypotheses are further described in detail in Section 2.2. Four fatigue algorithms were used: the maximum shear strain criterion (MSS), the Brown–Miller–Morrow criterion (BMM), the Morrow criterion (Morrow), and the Smith–Watson–Topper criterion (SWT). Figure 20 shows the number of logarithmic life repeats obtained for the fatigue algorithms used. The values presented are the minimal values of the logarithmic life repeats taken for each connection from the fatigue distribution (not shown).

## 4. Discussion

Comparative numerical studies between different engineering solutions, even if burdened by limited modeling error, provide sufficient insight into the engineering of lap connections to draw useful conclusions. In our study, we conducted parametric numerical studies on bolted joints with and without nuts (self-tapping connection) to compare the results between two practical solutions.

In case of the bolt with a nut, the bolt diameter parametrization showed that the higher the diameter, the greater the number of logarithmic life repeats, i.e., fatigue strength, as shown in Section 3.1.1. The logarithmic value for 10 mm was about 1700 while for 14 mm, it increased to more than 6000. The fatigue distribution shows that the most vulnerable points on the bolt are located in the corners, that is, in the places in which stress notches appear. The most critical area is the thread area of the bolt.

Similar effects were obtained for the nutless self-tapping bolt. The bolt diameter parametrization showed that the higher the diameter, the greater the number of logarithmic life repeats, as shown in Section 3.1.2. For instance, the logarithmic value for 8.2 mm was about 1050 while for 13 mm, it increased to no more than 1700. The fatigue distribution demonstrates that the most vulnerable points are located in the corners of the thread (next to the screw head), that is, in the places, in which stress notches appear.

Additionally, the material parameters of the bolts influence the fatigue strength. For the bolt with a nut, as shown in Section 3.1.2, it appears that the higher the yield strength of the material of the bolt, the lower the logarithmic life repeats. If the yield strength increases from 380 to 520 MPa, the logarithmic life repeats decrease almost two-fold from approximately 5200 to about 2700. The same effect was obtained for the nutless self-tapping bolt, as discussed in Section 3.2.2. Here, the drop was less significant: when the yield strength increased from 340 to 480 MPa, the decrease was approximately 41%.

For the bolt with a nut, the influence of the prestressing force on the fatigue strength was also numerically studied, as discussed in Section 3.1.3. The prestressing force was varied from 25 to 33 kN. The fatigue strength decreased with an increase in the prestressing force, decreasing by 40 % within the analyzed range, i.e., from 25 to 33 kN.

Moreover, for the nutless self-tapping bolt, the influence of the friction coefficient on the fatigue strength was numerically investigated, as discussed in Section 3.2.3. The friction coefficient varied from 0.05 to 0.15. The fatigue strength was almost constant with an increasing friction coefficient and the decrease observed was negligible.

In the end, how the use of different fatigue hypotheses, as discussed in Section 2.2, influences the value of the logarithmic life repeats was investigated. For the bolt with a nut, the values fluctuated from about 3900 for the SWT hypothesis (Equation (4)) to 5600 for the Morrow mean stress correction hypothesis (Equation (3)), as discussed in Section 3.1.4. For the self-tapping bolt without a nut, the value changed from about 1500 for the MSS hypothesis (Equation (1)) to 2400 for the Morrow mean stress correction hypothesis (Equation (3)), as discussed in Section 3.2.4.

The conducted analysis allows for the following comments:

- Both connection models were simplified by following the same assumptions; hence, the introduced simplifications had a similar effect on the final result of the connection fatigue strength;
- The connection with a self-tapping joint (nutless bolt) is similarly durable to the traditional connection with a bolt and a nut; and
- The connection with a self-tapping joint is more modern, faster, and easier to install due to the access required from only one side of the connection.

## 5. Conclusions

This paper presents fatigue analyses of screw connections of thin-walled structures. Stress and fatigue analyses were performed using the finite element method and analytical-empirical formulas. Two bolted connections were considered: the first was a screw with a nut and the second was a self-tapping screw without a nut. Both connections were subjected to parametric analysis. By selecting the most important parameters beforehand, their influence on the fatigue capacity was verified numerically. The parameters that gave the highest sensitivity of the connection in terms of the fatigue strength were indicated.

It appears that not only the geometric parameters influence the fatigue strength of connections, shown here on the bolt diameter, but also the steel yield strength or prestressing force. It should be underlined that considering different fatigue hypotheses gives meaningfully different estimations of the fatigue strength, but this is also reflected in the experimental studies. Both connections showed a similar fatigue strength.

## Figures and Tables

**Table 1:** Reference values of the parameters considered in the parametric study.

Parameter	Bolt with Nut	Nutless Bolt
Bolt diameter (mm)	12.0	13.2
Yield strength of the bolt (MPa)	450	340
Coefficient of friction between plates and bolt head (–)	–	0.10
Prestressing force (kN)	27	–
Fatigue hypothesis	Brown–Miller–Morrow criterion	Brown–Miller–Morrow criterion

## Data Availability

The data presented in this study are available on request from the corresponding author.
